# Use of Online Dietary Recalls among Older UK Adults: A Feasibility Study of an Online Dietary Assessment Tool

**DOI:** 10.3390/nu11071451

**Published:** 2019-06-27

**Authors:** Heather A. Ward, Heather McLellan, Chi Udeh-Momoh, Parthenia Giannakopoulou, Catherine Robb, Petra A. Wark, Lefkos Middleton

**Affiliations:** 1 Department of Epidemiology and Biostatistics, School of Public Health, Imperial College London, London W2 1PG, UK; 2 Department of Neuroepidemiology and Ageing, School of Public Health, Imperial College London, London W6 8RP, UK; 3 Faculty of Health and Life Sciences, Coventry University, Coventry CV1 5FB, UK; 4 Department of Primary Care and Public Health, School of Public Health, Imperial College London, London W6 8RP, UK

**Keywords:** myfood24, dietary recall, online, dietary assessment, older adults, cohort

## Abstract

This study examined the feasibility of including myfood24, an online 24-hour dietary recall tool, in a cohort studies of older adults. Participants (*n* = 319) were recruited during follow-up visits for the CHARIOT-Pro Sub-study, a prospective study of cognitively healthy adults aged 60–85 years at baseline. Email invitations were sent over three consecutive months, with weekly reminders. Multivariable regression models were applied to examine the number of recalls completed in relation to technology readiness (TR) scores and demographic characteristics. Ninety-four percent of people agreed to participate. Among participants, 67% completed at least one recall, and 48% completed two or more. Participants who completed multiple recalls reported higher self-confidence with technology and received a higher TR score than those who did not complete any recalls. A one-point higher TR score was associated with higher odds of completing three recalls compared to zero recalls (OR 1.70, 95% CI 0.96–3.01); this association was further attenuated after adjustment for demographic and other TR-related covariates (OR 1.35, 95% CI 0.63–2.88). This study demonstrates reasonable participation rates for a single myfood24 recall among older adults participating in a cohort study but suggests that further support may be required to obtain multiple recalls in this population.

## 1. Introduction

Unhealthy diets are a risk factor for developing chronic disease, including heart disease, stroke, cancer, diabetes and chronic lung disease [1]. As the global population ages, further research into the nature of diet-disease associations at different life stages is required. An ongoing challenge for nutritional epidemiology is accurate dietary assessment. Commonly used methods of dietary assessment within cohort studies include a semi-quantitative food frequency questionnaire (FFQ) or a 24-hour recall (24HR). In the latter, an inventory of all foods and beverages consumed in the past 24 h is taken. The advantages and limitations of each method have been well-described elsewhere [2]. For the 24HR, there is evidence that multiple recalls yield higher validity relative to biomarkers than single recalls [3,4], but the expense of multiple interviewer-administered recalls is prohibitive for most cohort studies. As a cost-effective alternative, online tools to collect self-administered 24HR have been developed. 

Several systems for collecting online 24HR have been developed internationally [5,6,7,8]. Overall, the online recalls have broadly demonstrated acceptable usability and validity [7,9,10,11]. However, there has been limited research to date on participation rates in online dietary assessment tools among older adults; in many studies, the results for adults over the age of 65 are pooled with those from younger adults. Touvier et al. included adults 48–75 years of age in a validation study of NutriNet-Santé, but reported on the overall participation rate rather than by age group [7]. A feasibility study of the Automated Self-Administered 24-hour dietary recall (ASA-24) among US adults 56–80 years of age highlighted the challenge of access: 60% invited reported no access to Internet, and adults 72–80 years of age were less likely than younger adults to have access to Internet/computer [12]. The ASA-24 (Canadian version) was used in a validation study among older adult Canadians, but participation results were not presented separately for relatively older and younger adults (over/under 65 years of age) [13]. Otherwise, feasibility studies have predominantly focused on adults younger than 70 years of age [14,15,16,17]. As such, the potential for collecting repeat online 24HRs for older adults remains unknown.

Myfood24 is an online 24HR dietary assessment tool aimed at the UK adult population [8]. The instrument has demonstrated feasibility in a clinical setting [18] and good validity among adults (<65 years of age) [11]. It therefore has the potential be a useful tool among older adults. However, previous studies of myfood24 included only a small subgroup of adults over 65 years of age (*n* = 5) [8]. The aim of the present study is to determine the feasibility of including myfood24 as a dietary assessment tool for cohort studies of older adults. 

## 2. Materials and Methods 

### 2.1. Participant Recruitment 

Participants for the myfood24 feasibility study were recruited from within the CHARIOT-Pro Sub-study (Imperial College London study site). CHARIOT-Pro is a prospective study of cognitively healthy adults (60–85 years of age) to characterize risk factors (health, lifestyle, cognitive, and biomedical) for the development of Alzheimer’s disease. In brief, CHARIOT-Pro participants were recruited from the CHARIOT register, a database of ~30,000 healthy research volunteers in West London, as well as through self-referral or referral via alternative means (e.g., family, friend, neighbour, etc.) [19]. Participants included the sub-study were identified as cognitively normal based on a zero score on the Clinical Dementia Rating Scale (CDR global score) and age- and education-adjusted RBANS score (Repeatable Battery for Assessment of Neuropsychological Status [20]) of less than 1.5 standard deviation below normal. Further details of the CHARIOT-Pro main study and sub-study, including recruitment, inclusion criteria, and schedule of visits, can be found online at ClinicalTrials.gov (Identifier: NCT02114372). This study has received National Research Ethics Services approval and internal Imperial College London Research Ethics, Joint Research Compliance Office approval. Prior to consenting to join the CHARIOT-Pro study in writing, participants were provided with a detailed study information sheet outlining study procedures, risks and benefits associated with participation. The present analysis is limited to data collected as part of the myfood24 feasibility study and participant demographic data (age, sex, education). 

All participants in the CHARIOT-Pro Sub-study were invited to join the myfood24 feasibility pilot study during a follow-up clinic visit between December 2016 and December 2018. Participants were given a brief demonstration of the myfood24 system by study research staff and advised that they would be asked to complete the recall at home on three occasions (once per month). Email invitations to complete myfood24 following the clinic visit were sent over three consecutive months, with a weekly reminder sent during the three weeks that followed each monthly invitation. The invitation and reminder days were the same for all participants within each monthly cycle, but the invitation and reminder days varied from month to month to accommodate weekday and weekend dietary variation. The initial invitation was sent during the first week of each month, and for approximately half of the data collection periods, the dietary recall data requested was from a weekend day.

### 2.2. Myfood24 

Myfood24 is an online 24-hour dietary recall tool targeted for use within large-scale epidemiological studies in the UK. The development of this instrument has been described previously [1,2], and a demo of the program can be viewed at www.myfood24.org. In brief, the myfood24 recall site allows participants to either search for and identify foods and beverages individually, or to compile a list of all foods and beverages consumed and then be guided through a search for further details of each item. Each item can be linked to an eating occasion (i.e., breakfast, lunch, evening dinner, snack, drink). Myfood24 contains a UK-specific database (Version 1.0, myfood24, University of Leeds, West Yorkshire, England) of 40,274 generic and branded food items, with associated food composition data for nutrient calculation [21]. Portion sizes are either entered manually or as multiples of the suggested average portion size. Myfood24 includes prompts for commonly forgotten foods and foods regularly consumed in combination with each other, warnings for eating occasions that do not contain any data, and a final review before submission. In the present feasibility study, participants were asked to report all foods and beverages consumed in the preceding day (midnight to midnight). Across the two-year data collection period, the day of week on which the email was sent varied to allow variation in weekday and weekend participation. However, the participants were able to choose when they initiated the recall. It was possible to pause the completion of myfood24, but after 24 h of inactivity the system would delete the incomplete recall. Participants were also invited to complete a brief user questionnaire to provide feedback on their experience with myfood24.

### 2.3. Technology Readiness Index

Upon recruitment to the myfood24 feasibility study, participants completed a technology readiness questionnaire (TRQ), which included general questions on confidence with technology, Internet ability and frequency of Internet access, previous completion of diet diaries, and previous completion of online diet diaries. In addition, the TRQ included an abbreviated Technology Readiness Index (TRI) designed for adults 50 years or older [22]. The original TRI is a 36-item scale assessing extent of agreement/disagreement with statements that reflect optimism, innovativeness, discomfort and security in user experiences with technology [23]; ten of these items were retained for the abbreviated version used in the present study. The overall technology readiness (TR) score for each respondent was obtained by averaging the scores of the four dimensions in the TRI: (Optimism) plus (Innovativeness) plus (6-Discomfort) plus (6-Insecurity); the possible range of TR scores was between one and five. The TRQ questionnaire was omitted from data collection for the first 89 participants due to an administrative error. 

### 2.4. Statistical Methods

Descriptive statistics were calculated as medians and interquartile ranges for continuous variables, and as frequencies and percentages for categorical variables. Multinomial logistic regression models were conducted to examine the TR scores in relation to the probability of completing myfood24 recalls (zero, one, two or three); the models were first adjusted for age at recruitment for myfood24 and sex, and then additionally for education (years), confidence with technology, Internet ability, and frequency of Internet use. Participants with missing data were excluded from statistical testing. 

The quality of the dietary data collected using the myfood24 recalls was evaluated by comparing total energy intake in relation to frequently used cut-offs for excessively extreme energy intakes (<500 kcal/day, >3500 kcal/day) [24].

## 3. Results

Three hundred and nineteen individuals were invited to participate in the myfood24 feasibility pilot, with consent obtained from 299 (93.7%) of those invited (Table 1). Among those who refused to consent (*n* = 20), lack of access to/skills with computers (*n* = 4) and lack of time were reported (*n* = 1); however, most refusers did not provide a reason (*n* = 15). Those who refused consent had a higher median age than those who consented (men: 75.3 years vs. 72.5 years, women 80.2 years vs. 71.3 years). Seventeen participants withdrew (5.3% of those who had consented; similar proportions of men and women). The reasons for withdrawal were: not recalling consent (*n* = 2), preference for paper copies (*n* = 1), reporting that the experience was too time consuming (*n* = 2) or difficult (*n* = 5), or no reason was given (*n* = 7). The median age of those who withdrew was higher than those who remained in the study (men: 77.0 and 71.0 years; women 77.4 and 71.5 years, respectively). 

Among the 282 participants who consented and remained in the study, 66.6% completed one or more recalls (Table 1). The number of recalls completed by men and women was broadly similar, with a slightly higher proportion of women completing three recalls (32.6% vs. 20.6%). Participants who completed the TRQ were similar in age, sex, education, and number of recalls completed to those who did not (Supplementary Table S1). 

Higher years of education were reported by those who completed two or more recalls (median 17 years) compared to those who completed no recalls (median 15.5 years) (Table 2). Adults ages 70 or greater comprised 58.9% of the study population; there was a relatively higher proportion of adults in this age category who did not complete any recalls (65.9%) compared to those who completed two or three recalls (54.8%) (Table 2). Participants who completed only one myfood24 recall reported the most prior experience with diet diaries (54.8%); in contrast, a higher proportion of those who completed multiple myfood24 recalls reported experience with online or smartphone records (20.4%). Participants who did not complete any recalls were most likely to rate their Internet ability as ‘Fair’ (23.6%) or ‘Good’ (31.9%). In contrast, those who completed two or three recalls were most likely to rate their Internet ability as ‘Very good’ (31.5%) or ‘Excellent’ (25.0%). Similarly, frequency of daily Internet access was higher among participants completing one recall (90.3%) or multiple recalls (92.6%) compared to zero recalls (81.9%). Participants who completed two or three recalls reported higher self-rated confidence with technology and received higher overall TR scores relative to those who completed one or zero recalls. 

In age- and sex-adjusted multinomial regression models, each additional point in TR score was associated with higher odds of completing three recalls compared to zero recalls (OR 1.70, 95% CI 0.96–3.01); this association was attenuated after adjustment for education and other aspects of the TR questionnaire (OR 1.35, 95% CI 0.63–2.88; Table 3). No association between the TRQ and the odds of completing one or two recalls was detected in the multivariable models. However, as a covariate in the multivariable models, education was associated with higher odds of completing two recalls [OR per year of education 1.15, 95% CI 1.00–1.33)], or three recalls [OR per year of education 1.14, 95% CI 1.01–1.29)], independent of age, sex, and characteristics from the technology readiness questionnaire. Age was not significantly associated with the number of recalls completed in either the simple or the multivariable analyses. 

Most recalls included energy values did not fall outside excessively extremes: 98.4% of values from the first recall, 99.2% of values from the second recall, and 97.3% of values from the third recall were within the range of 500 kcal to 3500 kcal. 

Ninety-four percent of users (*n* = 188) completing the brief user questionnaire reported that they did not require assistance to open and complete myfood24. They described the overall experience with myfood24 mostly as ‘some parts difficult, some parts easy’ (46.3%), ‘easy’ (28.2%) or ‘very easy’ (9.6%). A small proportion of participants described their experience as ‘difficult’ (12.8%) or ‘very difficult’ (2.7%). When asked if they preferred myfood24 or the FFQ administered in CHARIOT-PRO, a large proportion indicated no preference (28.7%) or that they had not yet completed the FFQ (37.0%). There was a slightly higher proportion of participants that preferred myfood24 over the FFQ (17.0% versus 13.3%). 

As informal qualitative feedback, study administrators reported that most common issues mentioned by participants included: (1) lack of homemade meal choices, (2) time consuming to enter entire recipes if food consumed not listed, (3) lack of specific brands, and (4) absence of food and beverage items consumed as part of a specialized diet. 

## 4. Discussion

This is the first feasibility study of online dietary assessment that has focused specifically on older adults. The overall participation rates were encouraging: two-thirds of participants completed as least one recall, and nearly half of those invited completed two or more recalls. However, previous research has shown greater precision when multiple online dietary recalls were completed [25], therefore efforts to identify ways to improve participation rates are warranted. In the present study, education was associated with higher likelihood of completing multiple recalls. Additionally, individuals who did not agree to participate or agreed but later withdrew from the study, were older than those who consented to participate and who remained in the study. Therefore, efforts to increase participation in the myfood24 instrument, or comparable tools, may be more effective if relatively older adults and adults with lower levels of education are better supported and/or followed-up. 

The potential importance of technology readiness in the context of completing online dietary recalls has been noted by other researchers [26]. In the present study, participants who did not complete any myfood24 recalls had different TRQ profiles relative to participants who had completed one or more myfood24 recalls: the former reported lower self-rated ability with the Internet and received lower overall scores for confidence with technology. However, the TRQ components were not statistically significant in multivariable models adjusting for age and education. It is possible that the absence of an association between TR score and completion of online recalls could be due to the relatively limited variation across education levels in the CHARIOT-Pro study. Participation bias in epidemiological research has been described previously: participants are more likely to be female, employed, and of relatively higher education and socioeconomic status [27]. This bias is larger among older adults, where those who participate in research studies are likely to be healthier and more active than same-aged adults who do not participate in research [28]. Participants in the present study were recruited from the CHARIOT-Pro Sub-study, which in itself recruited from the Chariot Register, a database populated through invitations to general practitioners in the West and Central London regions. In this study, response rates were higher among practices with a larger older population, lower socioeconomic disadvantage, and a higher proportion of white patients [19]. During the development of myfood24, self-rated technology confidence scores were lower among a small group of older adults (over the age of 65, *n* = 4) who completed myfood24 recalls compared to younger adults, but the scores were not evaluated in relation to completion of recalls [8]. In other fields of research, dimensions of the TRQ have been associated with the likelihood of adults complying with an e-booking system for medical appointments [29] and the likelihood of parents using an app to report child immunization [30]. The present analysis within a relatively highly educated and older population does not indicate that technological readiness is associated with later completion of online dietary recalls, but further research in populations with a wider range of demographic characteristics may be informative. 

The present study contributes to a relatively limited literature on electronic dietary recall feasibility among older adults; in many studies, the results for adults over the age of 65 are pooled with those from younger adults. In the NutriNet Santé study, overall participation was high: from an initial sample of 170 participants, only 15 did not complete any recalls, and five withdrew from the study; age-group specific participation statistics were not reported (age range 49–75 years) for relatively older and younger adults [7]. Feedback on the ASA24 online recall system among older Canadian adults was mixed: the majority indicated they felt confident (64.3%) and did not feel they needed tech support (77%) to complete the ASA24, but a large proportion of participants also found ASA24 to be unnecessarily complex (50.4%), cumbersome (43.4%) and not something they would use frequently (45.1%) [13]. The Canadian ASA24 study included adults of a wide range (45–87 years), and used both telephone and online recalls, therefore it is not possible to compare age-specific participation rates for online recalls to those obtained in the present analysis. Knowledge gaps remain regarding the approximate level of participation in online dietary recalls that could be expected in studies of adults aged 60 years and older. 

Obtaining high levels of participation for myfood24 in future studies of older adults may depend on multiple factors, including the cognitive function of participants and the level of training/support offered. Participants in the myfood24 feasibility study had undergone cognitive function assessment and screening prior to recruitment; for populations of older adults with poorer cognitive function, more accurate dietary data may be obtained through observer report rather than self-report [31,32]. In the present study, a brief demonstration of the myfood24 system was presented during the clinic visit. It is possible that additional support may have yielded higher participation rates for completing the recalls after the clinic visit; for example, completing the first recall in the clinic with assistance available. We are not aware of any studies that have been designed to compare participation rates of older adults in online dietary recalls under varying levels of support. Therefore the prospect of higher participation rates with greater in-person support is speculative, and evidence from other populations has indicated that the provision of assistance did not improve the validity of data from online recalls [33]. In addition to research into improving participation rates, the validity of myfood24 among older adults is yet to be established. A validation study of adults (18 to 65 years of age) collected a range of concentration, recovery, and predictive nutrient biomarkers and estimates of energy expenditure (accelerometry, calorimetry), and found that myfood24 yielded comparable estimates to the more costly interviewer-administered 24HR [11]. However, assessments of reporting accuracy among older adults can be affected by age-related changes in memory, body weight, vision and motor skills [32], also because older adults commonly under eat, which may be incorrectly perceived as underreporting [34]. Continued recognition of the potential differences between younger and older adults with respect to dietary assessment in general, and specifically within the context of online tools, is warranted. 

Strengths of the present analysis include a two-year data collection period, which allowed for a relatively large sample size for a feasibility study, and the use of screening tests to identify adults with poor cognitive function prior to recruitment in the present study. One limitation of the present study is that we did not collect feedback information from participants who agreed to participate but did not complete any recalls, which could have provided helpful insight into improving the participation rates for myfood24 or comparable online dietary recalls tools in future studies. We also did not use any qualitative assessment of feasibility, which would have complemented our findings by providing more in-depth insight into the barriers to and facilitators of participation to using an online 24 h dietary assessment tool in this user group. The plausibility of the energy intakes reported in the myfood24 recalls may have been overestimated as the cut-offs used may be more appropriate for a younger population with greater energy expenditure and energy intake. Characteristics of the CHARIOT register from which participants in the present study were drawn were described above; our results cannot be generalized to all older adults in the UK nor beyond. However, the present analysis provides an indication of the level of participation that could be expected among older adults who have agreed to contribute to health research. 

In conclusion, the present study demonstrated that the collection of at least one myfood24 dietary recall from the majority of participants in a cohort of older adults is feasible, and suggests that additional support and/or recruitment effort may be required to collect multiple recalls. Education level was associated with completing one or more recall, therefore the participation rate for this instrument in future studies may depend on the education level of the cohort. The field of electronic dietary assessment tools continues to develop. Ongoing assessment of the feasibility and validity of online dietary recalls will help inform the design of future epidemiological studies of older adults. 

## Figures and Tables

**Table 1:** Summary of study recruitment for the myfood24 feasibility study within the CHARIOT-Pro sub-study.

	Total	Women (*n* = 159)		Men (*n* = 160)	
Recruited (*n* = 319)	n (%)	n (%)	Age, years Median, (IQR)	n (%)	Age, years Median, (IQR)
Consented	299 (93.7)	150 (94.3)	71.3 (68.1–76.7)	149 (93.1)	72.5 (68.1–75.2)
Refused	20 (6.3)	9 (5.7)	80.2 (77.9–82.0)	11 (6.9)	75.3 (73.4–78.0)
Recalls ompleted (*n* = 282)		n (%)	Age, years Median, (IQR)	n (%)	Age, years Median, (IQR)
0	94 (33.3)	51(36.2)	71.9 (68.7–76.7)	43 (30.5)	72.9 (68.3–75.4)
1	54 (19.1)	23 (16.3)	71.3 (67.5–77.1)	31 (22.0)	70.7 (68.0–74.9)
2	59 (20.9)	21(14.9)	74.1 (67.6–77.4)	38 (27.0)	71.4 (68.9–74.6)
3	75 (26.6)	46 (32.6)	69.1 (67.3–74.2)	29 (20.6)	71.3 (66.9–74.3)

**Table 2:** Completion of zero vs. one or more myfood24 recalls: comparison of demographic and technology readiness characteristics.

	Completed Zero Recalls (*n* = 94)	Completed One Recall (*n* = 54)	Completed Two or Three Recalls (*n* = 134)
	n (%) †	n (%) ‡	n (%) §
Women	51 (54.3)	23 (42.6)	67 (50.0)
Education, years ††	15.5 (13–18)	16 (13–18)	17 (15–18)
Aged 70 years or older	62 (65.9)	31 (57.4)	73 (54.8)
Previously completed diet diary: no	41 (56.9)	14 (45.2)	56 (51.9)
Previously completed diet diary: yes	31 (43.1)	17 (54.8)	52 (48.1)
Previously completed online/smartphone diet record: no	63 (87.5)	25 (80.6)	86 (79.6)
Previously completed online/smartphone diet record: yes	9 (12.5)	6 (19.4)	22 (20.4)
Self-rated ability to use the Internet			
Poor	6 (8.3)	2 (6.5)	1 (0.9)
Fair	17 (23.6)	6 (19.4)	11 (10.2)
Good	23 (31.9)	9 (29.0)	35 (32.4)
Very good	14 (19.4)	8 (25.8)	34 (31.5)
Excellent	12 (16.7)	6 (19.4)	27 (25.0)
Frequency of using the Internet at home			
Less than once a week	3 (4.2)	2 (6.5)	2 (1.9)
1–6 times per week	10 (13.9)	1 (3.2)	6 (5.6)
Daily	59 (81.9)	28 (90.3)	100 (92.6)
	Median, IQR	Median, IQR	Median, IQR
Self-rated confidence with technology (scale 1–10)	7 (5–8)	7 (5–9)	8 (7–9)
TR score	3.2 (2.8–3.6)	3.2 (2.8–3.5)	3.5 (2.9–3.8)

† Missing data: education (*n* = 2); all aspects of TRQ (*n* = 22). ‡ Missing data: education (*n* = 5); all spects of TRQ (*n* = 23). § Missing data: education (*n* = 3); all aspects of TRQ (n = 26). †† median (interquartile range).

**Table 3:** Multinomial logistic regression analysis of TR scores in relation to the number of online recalls completed.

	OR	(95% CI)	*p*-Value
Model 1			
Technology readiness score *:			
One recall	0.98	(0.49–1.97)	0.96
Two recalls	1.40	(0.75–2.63)	0.29
Three recalls	1.70	(0.96–3.01)	0.068
Model 2			
Technology readiness score *:			
One recall	1.10	(0.44–2.75)	0.85
Two recalls	0.87	(0.37–2.03)	0.75
Three recalls	1.35	(0.63–2.8)	0.44

*N* = 210 due to missing data. * Reference group: zero recalls. Model 1 Age and sex adjusted. Model 2: Adjusted for age, sex, education (years), self-rated Internet ability, frequency of Internet use, self-rated confidence with technology.

## Supplementary Materials

The following are available online at https://www.mdpi.com/2072-6643/11/7/1451/s1, Table S1: Comparison of participants missing TRQ compared to those who completed the TRQ.
