# Supplementary material for: Use of Online Dietary Recalls among Older UK Adults: A Feasibility Study of an Online Dietary Assessment Tool
Source: Nutrients. 2019 Jun 27;11(7):1451. doi: 10.3390/nu11071451 (PMC6682978; doi:10.3390/nu11071451)
Supplement: Supplementary file 1 [file nutrients-11-01451-s001.pdf]

Supplementary Table S1: Comparison of participants missing TRQ compared to those who completed the TRQ

	TRQ missing (n=73)<br>median (interquartile range)	TRQ completed (n=209)<br>median (interquartile range)
Age at recruitment (years)	70.8 (67.3 – 74.9)	71.9 (68.1 – 75.4)
Education (years)	16.0 (13.0 – 18.0)	16.0 (14.0 – 18.0)
	n (%)	n (%)
Female	37 (50.7)	104 (49.8)
Number of recalls completed:		
0	23 (31.5)	71 (33.9)
1	23 (31.5)	31 (14.8)
2	15 (20.6)	44 (21.1)
3	12 (16.4)	63 (30.1)
